# Quantification of Retinal Nerve Fibre Layer Thickness on Optical Coherence Tomography with a Deep Learning Segmentation-Free Approach

**DOI:** 10.1038/s41598-019-57196-y

**Published:** 2020-01-15

**Authors:** Eduardo B. Mariottoni, Alessandro A. Jammal, Carla N. Urata, Samuel I. Berchuck, Atalie C. Thompson, Tais Estrela, Felipe A. Medeiros

**Affiliations:** 1 Vision, Imaging and Performance (VIP) Laboratory, Duke Eye Center, Duke University, Durham, NC United States of America; 2 Department of Ophthalmology, Federal University of São Paulo, São Paulo, Brazil; 3 Department of Statistical Science and Forge, Duke University, Durham, NC United States of America

**Keywords:** Eye diseases, Medical imaging

## Abstract

This study describes a segmentation-free deep learning (DL) algorithm for measuring retinal nerve fibre layer (RNFL) thickness on spectral-domain optical coherence tomography (SDOCT). The study included 25,285 B-scans from 1,338 eyes of 706 subjects. Training was done to predict RNFL thickness from raw unsegmented scans using conventional RNFL thickness measurements from good quality images as targets, forcing the DL algorithm to learn its own representation of RNFL. The algorithm was tested in three different sets: (1) images without segmentation errors or artefacts, (2) low-quality images with segmentation errors, and (3) images with other artefacts. In test set 1, segmentation-free RNFL predictions were highly correlated with conventional RNFL thickness (r = 0.983, P < 0.001). In test set 2, segmentation-free predictions had higher correlation with the best available estimate (tests with good quality taken in the same date) compared to those from the conventional algorithm (r = 0.972 vs. r = 0.829, respectively; P < 0.001). Segmentation-free predictions were also better in test set 3 (r = 0.940 vs. r = 0.640, P < 0.001). In conclusion, a novel segmentation-free algorithm to extract RNFL thickness performed similarly to the conventional method in good quality images and better in images with errors or other artefacts.

## Introduction

Glaucoma is a progressive optic neuropathy associated with characteristic changes to the optic disc and retinal nerve fibre layer (RNFL)^1^. In particular, RNFL assessment has been shown to be often the first detectable sign of glaucomatous damage^2–4^. RNFL defects may precede irreversible functional loss by several years and, therefore, assessment of the RNFL is an important tool for early diagnosis of the disease.

Spectral-domain optical coherence tomography (SDOCT) has allowed objective and quantitative evaluation of the RNFL. In order to provide quantitative measurements of the thicknesses of different retinal layers, current SDOCT software applies automated algorithms to segment the layers of the retina, including the RNFL. However, the accuracy of segmentation algorithms can be undermined by artefacts and other segmentation errors, which may be present in 19.9% to 46.3% of SDODT images^5,6^. Careful review of SDOCT imaging for segmentation errors is particularly crucial, since such errors can alter measurements and adversely impact clinical management^7^.

Although segmentation errors can be manually corrected, this is a time-consuming task and may be impractical in a busy clinical practice. However, the evolution of machine learning techniques and improved computational power have opened new approaches to expeditious quantification of structural damage on SDOCT images. In recent years, deep convolutional neural networks (CNN) have become the go-to algorithm for image classification, achieving near-human-level performance^8^. Characterized by the presence of several (i.e. deep) convolutional layers, CNN models extract low level features (e.g. corners, edges) in the initial layers that are hierarchically combined over numerous subsequent layers to extract more complex features, such as shapes and objects. This characteristic is especially useful for computer vision tasks such as layer segmentation and object detection, usually presenting better performance over standard imaging processing. For example, Devalla *et al*. recently developed a deep learning (DL) algorithm that can segment optic nerve head tissues with high accuracy relative to manual segmentation^9^.

Although it is helpful to have better segmentation algorithms, it would not eliminate the need for checking for errors. We hypothesized that a DL approach would allow estimation of the thickness of the RNFL without requiring segmentation of the retinal layers whatsoever. In addition, we hypothesized that a segmentation-free approach would outperform conventional segmentation algorithms in extracting thickness information from relatively low-quality scans prone to segmentation errors.

The purpose of the present study was to develop a novel DL neural network algorithm that can predict the RNFL thickness from raw SDOCT B-scans without requiring segmentation, and to evaluate its performance in both high-quality images as well as low-quality images that contain segmentation errors or other artefacts.

## Results

We developed a DL algorithm, trained on high-quality raw SDOCT B-scans, to predict the RNFL thickness values provided by the conventional software. We used a pre-trained residual neural network (ResNet34)^10^, further trained for fine-tuning. The training/validation dataset consisted of 13,262 images from 476 subjects and 897 unique eyes, split at the patient level for training (80% of the sample) and validation (20%).

Performance of the algorithm was evaluated in images from 441 eyes of 230 subjects, distributed between three different Test Sets. The Test Set 1 contained 11,010 images that were of high quality, without any artefacts or segmentation errors, according to the reading centre. Test Set 2 had 237 images with segmentation errors and Test Set 3 had 776 low-quality images with other artefacts. No subject from the test sets was present in either the training or validation datasets. Table 1 describes the demographic and clinical characteristics of the eyes and subjects in the study.

**Table 1:** Demographic and clinical characteristics of eyes and subjects in the study.

	Training	Validation	Test		
Subjects	380	96	230		
Eyes	718	179	441		
Heathy (%)	247 (34.4)	57 (31.8)	74 (16.8)		
Glaucoma suspects (%)	371 (51.7)	96 (53.6)	282 (64.0)		
Glaucoma (%)	100 (13.9)	26 (14.5)	85 (19.3)		
Age, years ± SD	60.8 ± 16.9	64.9 ± 15.7	66.4 ± 13.1		
Gender, female (%)	209 (56.03)	57 (60.64)	128 (55.65)		
Race, African American (%)	70 (18.4)	17 (17.71)	63 (27.39)		
			**Test Set 1**	**Test Set 2**	**Test Set 3**
Images (%)	10,520 (41.7)	2,742 (10.8)	11,010 (43.5)	237 (0.94)	776 (3.07)
Signal strength ± SD	26.87 (4.61)	26.62 (4.43)	27.07 (4.68)	26.66 (4.26)	20.90 (9.69)
Description	Images without segmentation errors or artefacts	Images without segmentation errors or artefacts	Images without segmentation errors or artefacts	Images with segmentation errors	Images with other artefacts

SD = standard deviation; SDOCT = Spectral-Domain Optical Coherence Tomography; RNFL = Retinal Nerve Fibre Layer.

### Deep learning algorithm performance

**Figure 1:**
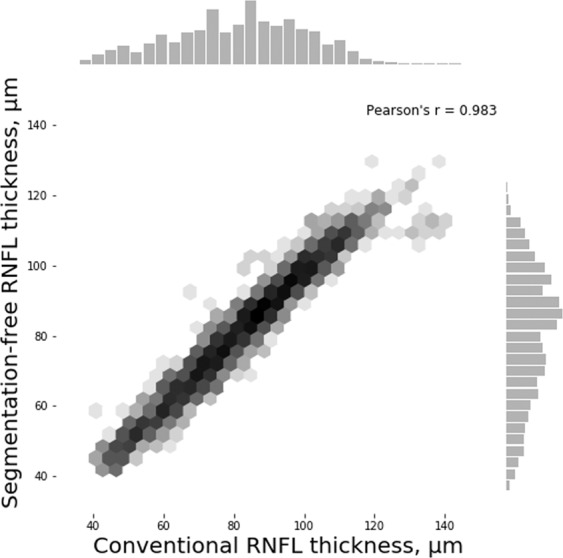
Scatterplot and histograms illustrating the relationship between the segmentation-free and conventional retinal nerve fibre layer (RNFL) thickness measurements from spectral domain optical coherence tomography (SDOCT) in good quality images from Test Set 1.

**Figure 2:**
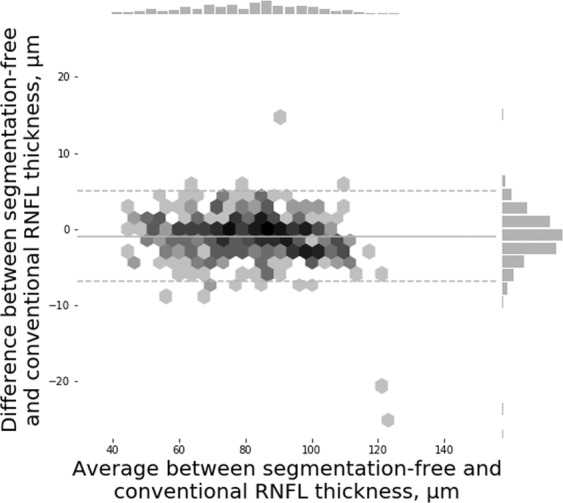
Bland-Altman plot for agreement between the segmentation-free and conventional retinal nerve fibre layer (RNFL) thickness from spectral domain optical coherence tomography (SDOCT) in good quality images (Test Set 1).

To evaluate the performance of the DL segmentation-free algorithm, the estimates of RNFL thickness given by the model were compared with the RNFL thickness measurements from the conventional segmentation-dependent algorithm in the images from Test Set 1 (high-quality images only). We found a strong correlation between DL segmentation-free RNFL estimates and the RNFL thickness obtained from the conventional segmentation-dependent algorithm (Pearson’s r = 0.983; P < 0.001), with a mean absolute error (MAE) of 2.41 µm. The performance of the algorithm was similar between healthy, suspected of having glaucoma and glaucoma subjects (Supplementary Table S1). Other factors, such as gender and race, also did not influence the performance of the algorithm (Supplementary Table S2). The 95% limits of agreement were −6.79 to 4.97 µm. Figure 1 illustrates the relationship between the segmentation-free and conventional global RNFL thickness values and Fig. 2 shows the Bland-Altman plot of the agreement between the two estimates for Test Set 1.

**Figure 3:**
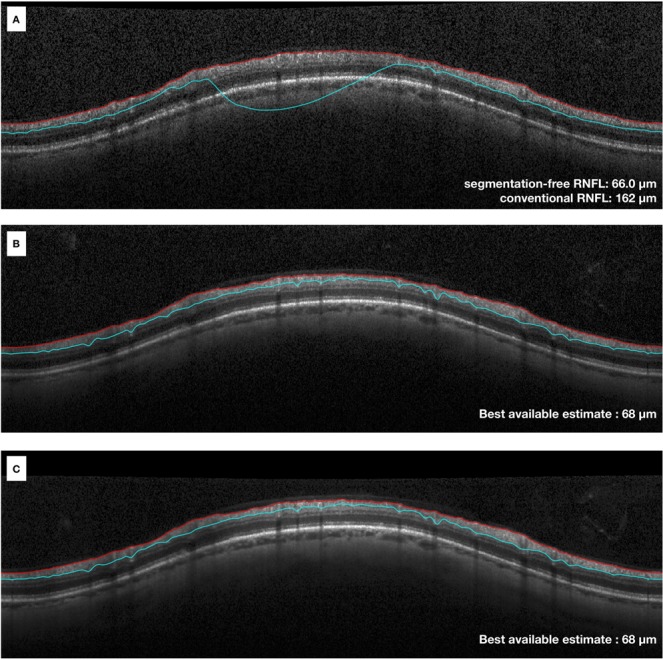
Example of B-scans taken at the same day for the same eye. Image (**A**) has an error in the delineation of the posterior boundary (blue line) of the retinal nerve fibre layer (RNFL), while images (**B,C**) are images with good quality and no segmentation errors. The best available estimate was 68 µm, calculated as the mean of the conventional RNFL thickness values from scans with good quality (scans B and C).

For Test Sets 2 and 3, composed of images with low quality, the RNFL thickness from the conventional algorithm could not be used as a reliable reference to evaluate the performance of the algorithm. We then used the average RNFL thickness of the scans with good quality of the same eye, taken at the same day, to provide the best available estimate (BAE; Fig. 3), used as a “ground-truth”. Both the DL segmentation-free predictions of RNFL thickness and the RNFL thickness measurements from the conventional segmentation-dependent algorithm were compared to the BAE.

**Figure 4:**
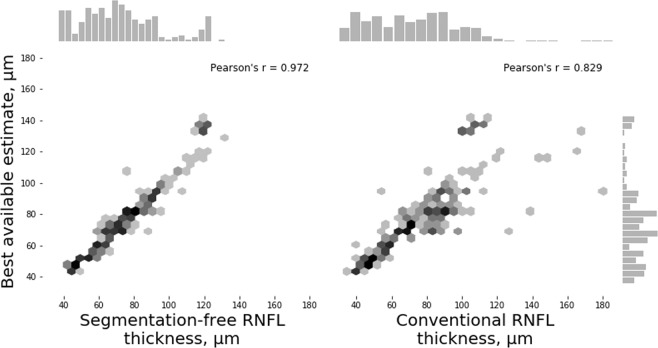
Relationship between the best available estimate and either the segmentation-free retinal nerve fibre layer (RNFL) or conventional RNFL thickness measurements in spectral domain optical coherence tomography (SDOCT) images with segmentation errors (Test Set 2).

In Test Set 2, segmentation-free RNFL thickness estimates showed a significantly stronger correlation with the BAE than those obtained with the conventional segmentation-dependent algorithm (r = 0.972 vs. r = 0.829, respectively; P < 0.001, William’s test of correlations). The MAE was 4.98 ± 5.85 µm for segmentation-free RNFL estimates and 8.59 ± 11.26 µm for conventional RNFL thickness estimates (P < 0.001, random effects mixed model). Figure 4 shows the relationship between RNFL thickness estimates obtained by the segmentation-free and conventional algorithms and the BAE.

**Figure 5:**
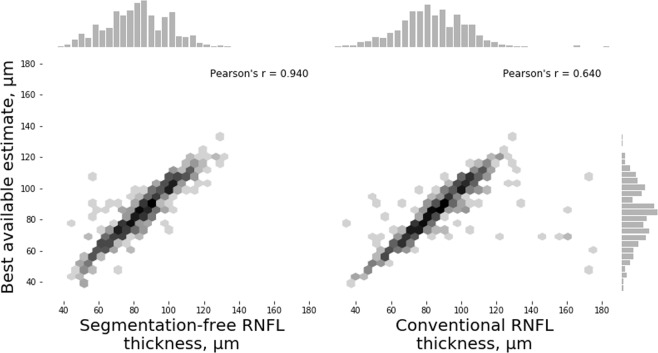
Relationship between the best available estimate and either the segmentation-free or conventional retinal nerve fibre layer (RNFL) thickness measurements in spectral domain optical coherence tomography (SDOCT) images with other artefacts (Test Set 3).

**Figure 6:**
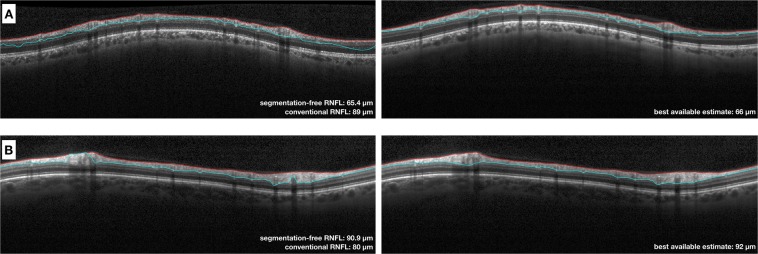
Examples of scans where the algorithm had a low error compared to the best available estimate (BAE). (**A**) Example of scan with segmentation errors that led to an overestimation of the global retinal nerve fibre layer (RNFL) thickness by the conventional algorithm (89 µm) compared to the BAE (66 µm, right column). The segmentation-free algorithm, however, was able to estimate the global RNFL thickness (65.4 µm) with a low error (approximately 0.6 µm). (**B**) Example of scan with a segmentation error that led to an underestimation of the global RNFL thickness by the conventional algorithm (80 µm) compared to the best available estimate (92 µm, right column). While the conventional algorithm underestimated the RNFL thickness by 12 µm, the segmentation-free algorithm prediction (90.9 µm) had an error of approximately 1.1 µm.

**Figure 7:**
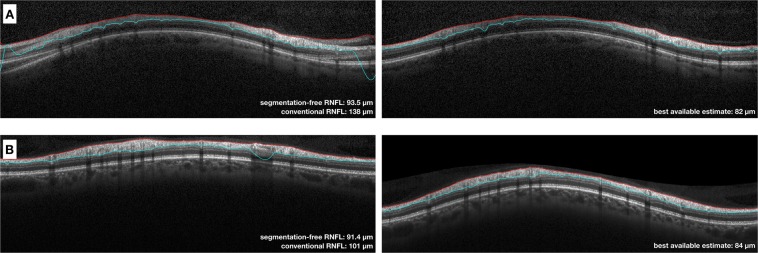
Examples of scans where the algorithm had a relatively high error compared to the best available estimate (BAE). (**A**) Example of scan with large segmentation errors and other artefacts. The segmentation-free algorithm estimated a global retinal nerve fibre layer (RNFL) thickness value of 93.5 µm. The BAE of global RNFL thickness from the good quality scan (right column) was 82 µm. Of note, the segmentation-free prediction was still much closer to the BAE compared to the conventional RNFL thickness measurement (138 µm). (**B**) Example of scan with a smaller segmentation error. There was a difference of approximately 7 µm between the segmentation-free estimate (91.4 µm) and the BAE (84 µm, right column). However, such difference was still smaller than that the 17 um difference seen between the conventional algorithm and the BAE.

In Test Set 3, segmentation-free RNFL thickness estimates also showed a significantly stronger correlation with the BAE than those obtained with the conventional segmentation-dependent algorithm (r = 0.940 vs. r = 0.640, respectively; P < 0.001, William’s test of correlations). The MAE was 3.94 ± 4.46 µm for segmentation-free RNFL and 5.06 ± 16.19 µm for conventional RNFL (P = 0.026, random effects mixed model). Figure 5 shows the relationship between RNFL thickness estimates from the segmentation-free and conventional algorithms and the BAE. Figure 6 shows examples of scans where the algorithm performed well, with a prediction error lower than the error of the conventional algorithm when compared to the BAE, and Fig. 7 show examples of scans where the segmentation-free algorithm had a relatively high error. Tables 2 and 3 summarize the MAE and the correlation coefficients, respectively, in all three test sets.

**Table 2:** Mean absolute error (MAE) of segmentation-free and conventional RNFL thickness in the three test sets in predicting the “ground truth” or best available RNFL thickness estimate.

	Number of images	MAE, µm		
		Segmentation-free algorithm	Conventional algorithm	P-value*
Test Set 1	11,010	2.41	—	—
Test Set 2	237	4.98	8.60	<0.001
Test Set 3	776	3.94	5.06	0.026

*P-value: random effects mixed models

MAE = mean absolute error.

**Table 3:** Correlation coefficients of segmentation-free and conventional RNFL thickness with ground truth or best available estimates in the three test sets.

	Number of images	Correlation coefficients		
		Segmentation-free algorithm	Conventional algorithm	P-value*
Test Set 1	11,010	0.983	—	—
Test Set 2	237	0.972	0.829	<0.001
Test Set 3	776	0.940	0.640	<0.001

^*^P-value: William’s test of correlations.

## Discussion

In the present study, we developed a segmentation-free DL algorithm capable of estimating global RNFL thickness from raw B-scans. The algorithm’s estimates of RNFL thickness were highly correlated with the RNFL thickness measurements provided by the conventional software in good quality images without segmentation errors or artefacts. In the two test sets that contained SDOCT images with segmentation errors or other artefacts, segmentation-free RNFL thickness estimates were much closer to the ground-truth estimates for RNFL thickness than those obtained by the conventional segmentation-dependent algorithm.

Our findings suggest that the segmentation-free DL algorithm can provide robust RNFL thickness estimates both in good quality images as well as in those that are prone to segmentation errors. Such algorithm could be helpful in clinical practice for assessing RNFL thickness without the need for segmentation, eliminating the time-consuming task of checking and manually correcting the segmentation of retinal layers. In addition, it could find use in providing a reference measurement to be used for flagging when estimates obtained using the conventional segmentation seem too discrepant from those obtained without segmentation, a situation that would highly suggest presence of significant segmentation errors.

Artefacts and segmentation errors are highly prevalent on SDOCT and result in erroneous estimates of RNFL thickness^5–7,11,12^. While Asrani *et al*. identified artefacts in 19.9% of RNFL circle scans^5^, Liu *et al*. found at least one artefact in 46.3% of the 2,313 scans analysed in their study^6^. Mansberger and colleagues demonstrated that manual refinement changed the glaucoma classification of 8.5% of scans, 7.7% to a less severe and 0.8% to a more severe classification. Also, automated segmentation resulted in a global RNFL thickness that was 1.6 µm thinner than the “true” thickness as determined by manual refinement. The difference between the automated and “true” thickness increased with older age, thinner RNFL thickness, and lower scan quality^7^.

Many groups have tried to develop automated algorithms for retinal segmentation in macular^13–15^ and optic nerve head scans^9,16^. Devalla and colleagues developed a DL algorithm for retinal segmentation in optic nerve head scans^9^. The algorithm achieved good accuracy when compared to manual segmentation performed by two human graders. The resulting RNFL thickness provided by this algorithm had an error of 8.85 ± 3.40% and 9.01 ± 4.20% when compared to each grader, while the graders had an error of 5.94 ± 2.30% between each other. Rather than trying to improve the segmentation process, we trained a DL algorithm to predict the global RNFL thickness from raw B-scan images without requiring segmentation. This was accomplished by providing reliable global RNFL thickness estimates from conventional segmentation software as the target value. This forced the DL algorithm to come up with its own way of finding how to estimate RNFL thickness. The idea was to avoid using prespecified thresholds or filters as usually performed in conventional segmentation algorithms. We hypothesized that such approach would provide a more robust way of estimating RNFL thickness. As expected, when applied to high-quality images from Test Set 1, the DL estimates of RNFL thickness were at least as good as those provided by the conventional segmentation algorithm, with a MAE of only 2.41 μm, which translates into an error percentage of only 3.0 ± 2.7%, lower than that achieved in Devalla’s study. Importantly, the MAE of the DL predictions did not exceed the instrument’s reported test-retest variability, which ranges from 4.02 to 5.30 µm according to the literature^17–21^.

The most important aspect of our work was to demonstrate that the segmentation-free DL algorithm would be able to provide reliable estimates of RNFL thickness in images where the conventional segmentation algorithm would fail. For this purpose, we identified samples of images (Test Sets 2 and 3) that contained segmentation errors or other artefacts. One important challenge was how to evaluate the performance of the DL estimates in comparison to the conventional algorithm. As the RNFL thicknesses provided by the conventional segmentation software in these cases would likely be inaccurate, they could not be used as a reliable value for true reference. Instead, the global RNFL thickness from scans with good quality taken from the same eye on the same day were averaged to provide a BAE, or “ground truth”. We found that the MAE in relation to the “ground truth” for the conventional method was more than 70% higher than that for the DL algorithm in images with segmentation errors, and almost 30% higher in images with other artefacts. Furthermore, the correlation with the BAE was higher for the segmentation-free DL RNFL thickness than for the RNFL thickness obtained from the conventional segmentation software. These findings suggest that application of the DL algorithm may improve the utility of relatively low-quality scans acquired in routine clinical practice.

This study has limitations. Since this was a supervised DL algorithm, it was trained to be only as good as the target values used in the training process. In our study, the target values were the RNFL thickness measurements in high-quality images according to the conventional SDOCT software. To guarantee proper target values for the training process, only high-quality images that had correctly positioned segmentation lines were used. Although RNFL thickness estimates provided by the conventional algorithm in these images are likely to be accurate, they can only be as good as the conventional segmentation approach allows. The estimates may still be influenced by blood vessels embedded in the RNFL, for example. Furthermore, the presence of segmentation lines allows the examiner to manually revise for errors, while in a segmentation-free approach this is not possible. As another limitation, in this proof-of-concept study the DL algorithm was trained to predict only the global RNFL thickness from the SDOCT B-scans. Future work should concentrate on obtaining segmentation-free RNFL estimates at each individual location on the B-scan or within predefined sectors. It will also be important in future studies to assess the performance of the DL segmentation-free algorithm in populations with other characteristics, such as eyes with high myopia. In addition, it will be important to assess how the RNFL thickness estimates perform in detecting glaucoma progression and estimating rates of change in a longitudinal setting.

In conclusion, we developed a novel segmentation-free DL algorithm that can predict accurate RNFL thickness estimates from raw SDOCT B-scan images, without requiring segmentation of the retinal layers. The algorithm provided reliable estimates both in high-quality images as well as in images prone to segmentation errors and other artefacts. Application of this algorithm in clinical practice may improve the utility of low-quality SDOCT scans and may help decrease errors in the interpretation of RNFL measurements.

## Methods

The data in this study is a cross-sectional sample from the Duke Glaucoma Repository, a database of electronic medical and research records at the Vision, Imaging, and Performance Laboratory at Duke University. The Institutional Review Board approved this study, and a waiver of informed consent was granted due to the retrospective nature of this work. All methods adhered to the tenets of the Declaration of Helsinki for research involving human subjects and the study was conducted in accordance with regulations of the Health Insurance Portability and Accountability Act (HIPAA).

The database included information from comprehensive ophthalmologic examinations collected during follow-up, such as medical history, visual acuity, intraocular pressure, slit-lamp biomicroscopy, gonioscopy, and dilated slit-lamp funduscopic examinations. In addition, mean deviation (MD) from standard automated perimetry (SAP) with the 24-2 Swedish interactive threshold algorithm (Carl Zeiss Meditec, Inc., Dublin, CA) was collected. Visual fields were retained if they met the following reliability criteria: fewer than 33% fixation losses and fewer than 15% false-positive errors. Spectralis SDOCT (Software version 5.4.7.0, Heidelberg Engineering, GmbH, Dossenheim, Germany) imaging was also performed.

Diagnosis of glaucoma was defined based on the presence of abnormal repeatable visual field loss in SAP (pattern standard deviation <5% or glaucoma hemifield test outside normal limits) and signs of glaucomatous optic neuropathy as based on records of slit-lamp fundus examination. Glaucoma suspects were those with suspicious appearance of the optic disc on slit-lamp fundus examination, but without repeatable visual field loss in SAP. Healthy subjects were required to have a normal optic disc appearance on slit-lamp fundus examination in both eyes as well as no history of elevated intraocular pressure and normal SAP results.

### Spectral-domain optical coherence tomography

The Spectralis device uses a dual-beam SDOCT and a confocal laser-scanning ophthalmoscope that employs a super luminescent diode light with a wavelength of 870 nm as well as an infrared scan to provide simultaneous images of ocular microstructures. For this study, a Spectralis RNFL circle scan was used to acquire 1,536 A-scan points within a 3.45-mm circle centred on the optic disc. Both corneal curvature and axial length measurements were entered into the instrument software to ensure appropriate scaling of all measurements. Also, the device’s eye-tracker was used to adjust for eye movements during image acquisition.

Segmentation lines were delineated by the conventional software in the Spectralis SDOCT, including the inner and outer RNFL boundaries, or the inner limiting membrane and the inner plexiform layer, respectively. A reading centre assessed the quality of each scan by evaluating the following characteristics: signal strength, illumination, scan centration, clipped scans, segmentation errors, floaters, and other pathologies or other artefacts. Any misalignment was considered a segmentation error. The average peripapillary RNFL thickness corresponding to the 360-degree measurement was automatically calculated by the SDOCT software^22^.

### Image processing and development of the deep learning algorithm

A DL algorithm was trained to predict global RNFL thickness using raw B-scans (i.e., without automated delineation of the RNFL). By training the model with unsegmented images, the algorithm was forced to adjust its filters and combination of filters in a unique and unrestricted way, in order to identify all available features in the image that could be relevant to predict the RNFL thickness measurement. To ensure that the algorithm was trained using reliable RNFL measurements, the training/validation dataset consisted only of high-quality raw B-scan images that contained no segmentation errors or artefacts, as determined by a reading centre. The dataset was randomly split at the patient level into training (80%) and validation (20%), thus preventing leakage from subject-related characteristics.

For training the algorithm, the images were down-sampled to a size of 496 × 496 pixels and pixel values were scaled to range from 0 to 1. The training data was augmented by performing the following image transformations: random lighting (i.e. subtle changes in image balance and contrast), random rotation (i.e. rotations of up to 10 degrees in the image) and flipping the image horizontally. This process increased the heterogeneity of the images, thus reducing the possibility of overfitting and enabling the algorithm to learn the most relevant features in each image.

We used the Residual deep neural Network (ResNet34) architecture^10^, which had been previously trained on the ImageNet dataset^23^. The ResNet allows rapid training of deep CNNs through identity shortcut connections that skip one or more layers and decrease the vanishing gradient issue during training^10^. We adapted its architecture to provide a continuous number by replacing the last layer of the network with a layer with a single linear output. Training was performed by first unfreezing the last 2 layers, and then subsequent model fine-tuning was performed with all of the layers unfrozen and using differential learning rates. The DL algorithm was trained with minibatch gradient descent of size 64 and Adam optimizer^24,25^. The best learning rates were found using the cyclical learning method with stochastic gradient descents with restarts.

After data processing and training were completed, the DL algorithm was used to predict the global RNFL thickness from raw SDOCT B-scan images without segmentation lines (i.e., segmentation-free RNFL) from images in the three test sets.

### Performance assessment

We assessed performance of the algorithm in three distinct test sets. No patient from the training or validation set was present in any of the test sets. Test Set 1 consisted of images without segmentation errors or artefacts according to human gradings. Meanwhile, Test Sets 2 and 3 had images with errors; in particular, Test Set 2 included images with segmentation errors that could not be manually corrected, and Test Set 3 contained images with other artefacts, such as illumination or centration problems, clipped scans, and low-quality score. For each of the images in Test Sets 2 and 3, there was at least one accompanying image from the same eye and day with good quality. The RNFL thickness from good quality SDOCT scans provided a BAE that served as the “ground truth” for evaluation of the RNFL estimates from low-quality images. If more than one of these good quality images existed, then an average of the RNFL thicknesses was calculated as the BAE. Figure 1 shows examples of scans with good and low-quality of the same eye, taken at the same day.

The study had two main hypotheses:

**Hypothesis 1**: In good-quality images, the segmentation-free algorithm would perform similarly to the conventional segmentation in predicting RNFL thickness measurements. To test this hypothesis, RNFL thickness measurements were extracted from raw B scans using the DL segmentation-free algorithm in Test Set 1, which contained only good quality images. MAE and Pearson’s correlation coefficients between the segmentation-free and conventional segmentation algorithm were calculated, as well as a Bland-Altman analysis modified to account for repeated measures^26,27^.

**Hypothesis 2**: In low-quality images or images with segmentation error, the DL segmentation-free algorithm would provide RNFL thickness measurements that would be closer to the “ground-truth” than those provided by the conventional segmentation algorithm. To test this hypothesis, we used Test Sets 2 and 3. The “ground-truth” was the BAE described above. MAE and Pearson’s correlation coefficients were calculated between the BAE and the estimates derived from the segmentation-free and conventional algorithm. MAEs for the conventional and segmentation-free algorithms were compared using a random effects mixed model, that accounted for variability across patients and eyes^28^. Pearson correlation coefficients were compared using a hypothesis test for comparing two correlations with a common variable^29^.

All statistical analyses were performed using Stata (version 15, StataCorp LP, College Station, TX). The alpha level (type I error) was set at 0.05.

## Supplementary information

Supplementary tables S1 and S2.
