# Supplementary material for: Quantification of Retinal Nerve Fibre Layer Thickness on Optical Coherence Tomography with a Deep Learning Segmentation-Free Approach
Source: Sci Rep. 2020 Jan 15;10:402. doi: 10.1038/s41598-019-57196-y (PMC6962147; doi:10.1038/s41598-019-57196-y)
Supplement: Supplementary file 1 — Supplementary tables S1 and S2. [file 41598_2019_57196_MOESM1_ESM.docx]

**Quantification of Retinal Nerve Fibre Layer Thickness on Optical Coherence Tomography with a Deep Learning Segmentation-Free Approach**

**Eduardo B. Mariottoni, MD,^1,2^ Alessandro A. Jammal, MD,^1^ Carla N. Urata, MD,^1^ Samuel I. Berchuck, PhD,^1,3^ Atalie C. Thompson, MD, MPH,^1^ Tais Estrela, MD,^1^ Felipe A. Medeiros, MD, PhD^1^**

1. Vision, Imaging and Performance (VIP) Laboratory, Duke Eye Center, Duke University, Durham, NC.
2. Department of Ophthalmology, Federal University of São Paulo, São Paulo, Brazil.
3. Department of Statistical Science and Forge, Duke University, Durham, NC.

*felipe.medeiros@duke.edu

**Supplementary Table S1**. Mean absolute error (MAE) of segmentation-free thickness and correlation coefficients with conventional retinal nerve fibre layer thickness in good quality images (test set 1), grouped by diagnosis.

|  | **Number of images** | **MAE, µm** | **Correlation coefficients** | **95% confidence interval*** |
| --- | --- | --- | --- | --- |
| **Healthy** | 1,912 | 2.40 | 0.955 | 0.939 – 0.972 |
| **Glaucoma suspects** | 6,673 | 2.47 | 0.974 | 0.967 – 0.981 |
| **Glaucoma** | 2,425 | 2.25 | 0.978 | 0.967 – 0.988 |

MAE = mean absolute error

Comparisons of absolute errors were performed using a random effects mixed model and were not significant: Healthy vs Glaucoma suspects: p = .807; Healthy vs Glaucoma: p = .915; Glaucoma vs Glaucoma suspects: p = .875.

95% confidence interval of correlation coefficients were calculated using a bootstrap method.

**Supplementary Table S2**. Mean absolute error (MAE) of segmentation-free thickness and correlation coefficients with conventional retinal nerve fibre layer thickness in good quality images (test set 1), grouped by gender and race.

|  | **Number of images** | **MAE, µm** | **Correlation coefficients** | **95% confidence interval** |
| --- | --- | --- | --- | --- |
| **Gender,** |  |  |  |  |
| **Male** | 5,958 | 2.53 | 0.981 | 0.975 - 0.986 |
| **Female** | 5,052 | 2.31 | 0.985 | 0.980 - 0.990 |
| **Race,** |  |  |  |  |
| **Caucasians** | 6,733 | 2.39 | 0.981 | 0.975 - 0.986 |
| **African Americans** | 3,542 | 2.56 | 0.983 | 0.978 - 0.988 |
| **Other races** | 735 | 1.87 | 0.995 | 0.991 - 0.999 |

MAE = mean absolute error

Comparisons of absolute errors were performed using a random effects mixed model and were not significant: Male vs Female: p = .772; Caucasians vs African Americans: p = .569; Caucasians vs Other races: p = .656; African Americans vs Other races: p = .469.

95% confidence interval of correlation coefficients were calculated using a bootstrap method.
